# Trans-Atlantic exchanges have shaped the population structure of the Lyme disease agent *Borrelia burgdorferi* sensu stricto

**DOI:** 10.1038/srep22794

**Published:** 2016-03-09

**Authors:** S. Castillo-Ramírez, V. Fingerle, S. Jungnick, R. K. Straubinger, S. Krebs, H. Blum, D. M. Meinel, H. Hofmann, P. Guertler, A. Sing, G. Margos

**Affiliations:** 1 Programa de Genómica Evolutiva, Centro de Ciencias Genómicas, Universidad Nacional Autónoma de México, Apartado Postal 565-A, CP 62210, Cuernavaca, Morelos, México; 2 National Reference Center for Borreliosis at the Bavarian Health and Food Safety Authority, Veterinärstr. 2, 85764 Oberschleissheim, Germany; 3 LMU Munich, Department of Infection and Zoonoses, Veterinärstr. 13, 80539 Munich, Germany; 4 LMU Munich, Gene Centre, Lafuga, Feodor-Lynen-Strasse 25, 81377 Munich, Germany; 5 TU Munich, Klinik für Dermatologie and Allergologie, 80802 Munich, Germany

## Abstract

The origin and population structure of *Borrelia burgdorferi* sensu stricto (s.s.), the agent of Lyme disease, remain obscure. This tick-transmitted bacterial species occurs in both North America and Europe. We sequenced 17 European isolates (representing the most frequently found sequence types in Europe) and compared these with 17 North American strains. We show that trans-Atlantic exchanges have occurred in the evolutionary history of this species and that a European origin of *B. burgdorferi* s.s. is marginally more likely than a USA origin. The data further suggest that some European human patients may have acquired their infection in North America. We found three distinct genetically differentiated groups: i) the outgroup species *Borrelia bissettii*, ii) two divergent strains from Europe, and iii) a group composed of strains from both the USA and Europe. Phylogenetic analysis indicated that different genotypes were likely to have been introduced several times into the same area. Our results demonstrate that irrespective of whether *B. burgdorferi* s.s. originated in Europe or the USA, later trans-Atlantic exchange(s) have occurred and have shaped the population structure of this genospecies. This study clearly shows the utility of next generation sequencing to obtain a better understanding of the phylogeography of this bacterial species.

Genome sequencing has drastically changed our understanding of microbial microevolution over the past decades. This is particularly the case for bacteria causing infectious diseases. Furthermore, the advent of next-generation sequencing (NGS) has allowed scientists to handle large population samples and to address a variety of biological questions concerning the evolution and ecology of bacterial pathogens. For example, NGS data has been used to study pathogen evolution within a single patient^1^, transmission chains within a single hospital^2^,^3^ and clonal diversification over time and space^4^,^5^. Without any doubt the unprecedented resolution provided by NGS is changing the way in which evolutionary biology and molecular epidemiology studies are conducted^4^,^5^,^6^.

*Borrelia burgdorferi* sensu stricto, a tick-borne spirochete bacterium, belongs to the species complex *B. burgdorferi* sensu lato (s.l.) that now comprises more than 20 accepted and proposed species of which five are regularly isolated from human patients^7^,^8^. The species that cause Lyme Borreliosis (LB) (also called Lyme disease) include *B. burgdorferi* s.s., *B. afzelii*, *B. spielmanii*, *B. garinii* and *B*. *bavariensis*. LB can be regarded as a multi-systemic disease, because it presents different symptoms in patients, such as erythema migrans (EM), arthritis, neurological manifestations (neuroborreliosis) or lymphocytoma^9^,^10^,^11^. So far, *B. burgdorferi* s.s. is the only human pathogenic *Borrelia* species of the Lyme borreliosis group of spirochetes in North America^9^ and symptoms described in the USA differ somewhat from those in Europe^11^,^12^,^13^.

LB is a zoonotic vector-borne disease, hence, the natural transmission cycles of these bacteria depend on vertebrate reservoir hosts and vector ticks of the *Ixodes persulcatus* species complex^14^,^15^. This ecological dependence determines the geographic distribution of the bacteria (and the disease) to the temperate zones of the northern hemisphere (roughly from latitude 40 degree - 60 degree N). Within this geographic range of vector and host occurrence, the distribution of *Borrelia* species is not uniform. Some *Borrelia* species (e.g. *B. garinii*, *B. afzelii* and *B. bavariensis)* are only found in Eurasia, whereas *B. burgdorferi* s.s. occurs on both sides of the Atlantic Ocean^16^.

Understanding the movement or spread of bacterial pathogens is important for epidemiological surveillance and monitoring and to predict the spread of such pathogens into new geographic regions. Researchers have used *B. burgdorferi* s.s. to understand the movement of these bacteria within endemic areas but also into new geographic regions^17^,^18^,^19^,^20^,^21^,^22^,^23^,^24^. Depending on the molecular markers used, the origin of the genospecies *B. burgdorferi* s.s. has been suggested to be in the USA (*ospC*^23^,^25^) or Europe (multilocus sequence typing [MLST]^20^). Furthermore, population level studies using *ospC* suggested that *ospC* type A strains crossed the Atlantic Ocean approximately 200 years ago^23^. Although *B. burgdorferi* s.s. was one of the first bacterial species being sequenced^26^, completed genomes are available only for 17 isolates, mainly originate from the USA (14 out of 17)^27^,^28^,^29^,^30^.

In this study, we exploit the high resolution of NGS to obtain a better understanding of the relatedness of populations from Europe and the USA, with a particular view on human patient isolates from Europe, and to re-address the question of the origin of *B. burgdorferi* s.s.. We included in our analysis some previously sequenced strains to create a more comprehensive data set for this species in terms of genome sequences and geographic origin.

## Results

### The data set and genome sequencing

We sequenced 17 European isolates (Table 1) using the Illumina MiSeq technology to compare them to fully sequenced genomes available in GenBank, the majority of these strains originated from the USA (see http://borreliabase.org/). As we aimed to infer the evolutionary history of strains, we chose to analyse the main chromosome, which contains the genetic information of the core genome. In general, the reads mapped uniformly to the B31 main linear chromosome with more than 90 percent of the chromosome covered. Mean median coverage was 221× (minimum coverage 34× for sample PLue, maximum coverage 419× for sample PAli) (Supplementary Table 1). Regardless of coverage, we observed an alignment gap in the region corresponding to 435 kbp to 442 kbp of B31 (with some variation between strains) for all strains. In B31 this region contains the 5S–23S ribosomal DNA which is duplicated in LB spirochetes^31^ and this duplication evidently presents a problem for proper mapping of NGS sequence reads. We included in our analyses 17 genome sequences that are publicly available (Table 2); most of them are from the USA (n = 14) while three are from Europe (Bol26, SV1, and ZS7) and the closely related species *B. bissettii* strain DN127 was used as an outgroup. We used progressive Mauve^32^ to align the chromosomal genomes of our newly sequenced strains as well as those from the previously published strains. We found 92 locally colinear blocks (LCBs), eight of them contain DNA conserved in all the strains and were used for downstream analysis; these eight LCBs are described in Supplementary Table 2. Of note, we observed similar levels of genetic variation across the eight LCBs (see Supplementary Table 2). In summary, we compiled a data set of 35 strains that contained isolates from both Europe and the USA (including an outgroup).

### Three genetically differentiated groups

**Figure 1:**
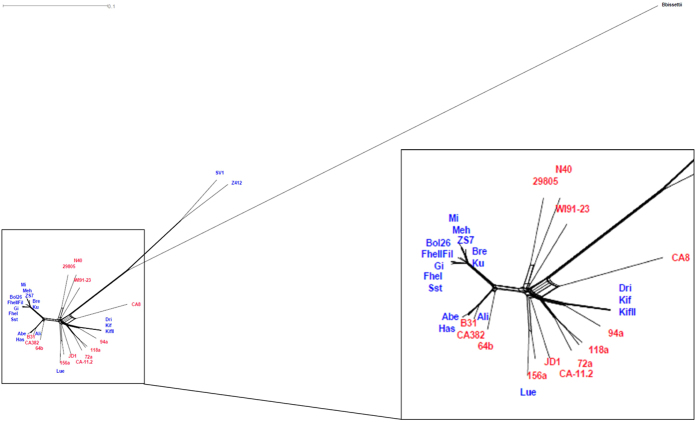
Neighbor Net of the 34 isolates. Neighbor Net based on all the SNPs, the uncorrected p distance was used and the network was drawn employing the equal angle method. In red are the strains from the USA, whereas blue labels depict the European strains. Considerable reticulation can be observed in the network (see inlet), which is compatible with the notion that recombination has affected this data set. The Phi test for detecting recombination was conducted and significant signals for recombination were found (p < 0.001).

**Figure 2:**
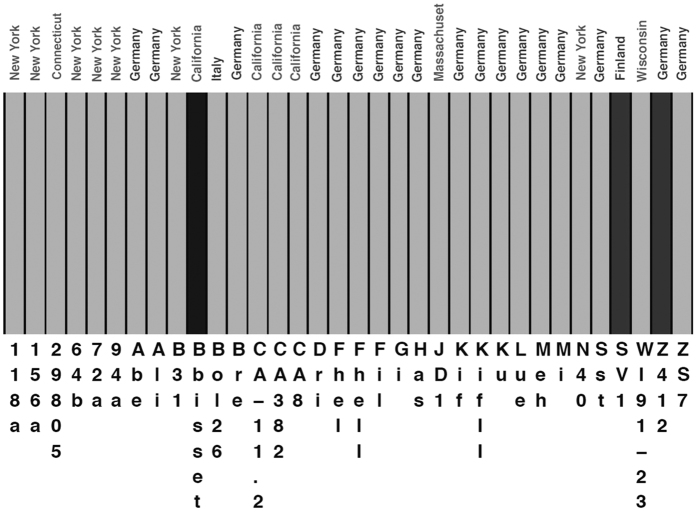
Mixture and Admixture analyses of the data set. Mixture and Admixture analyses were conducted through BAPS. Each color represents one of three genetically differentiated groups and each vertical colored bar corresponds to one isolate. The color coding is as follows: black, BAPS group 3; dark grey, BAPS group 2; light grey, BAPS group 1. No evidence for admixture between groups was found.

To visualize the genetic relationships among the strains, we first employed a neighbor net (Fig. 1), as this approach accounts for the possibility of recombination. It is known that significant amounts of variation on plasmids of *B. burgdorferi* s.s. have arisen by recombination^33^, and our analysis showed that some recombination seems to occur on the main chromosome (Fig. 1). As would be expected if recombination occurs, the network showed some reticulation reflecting conflicting phylogenetic signals; corroborating this result, the PhiTest gave significant signals for recombination (p < 0.001). Neither the European strains (blue) nor the American strains (red) formed a monophyletic group (Fig. 1). Actually, there were several clades that contained both European and American strains. Strains SV1 and Z41293 (which were both isolated from *I. ricinus* ticks) clustered together and were well separated from the rest of the strains, suggesting that these two strains comprised divergent strains of *B. burgdorferi* s.s.. All *B. burgdorferi* s.s. strains were clearly differentiated from the outgroup and although SV1 and Z41293 were considerably separated from the other *B. burgdorferi* s.s. strains, they were much closer to the rest of the *B. burgdorferi* s.s. strains than to the *B. bissettii* outgroup. To further study the genetic relationships of these strains we used the Bayesian Analysis of Population Structure software (BAPS)^34^. Three genetically differentiated groups were found (Fig. 2). The smallest group (BAPS group 3) contained exclusively the outgroup strain (black bar) and another small group (BAPS group 2) had the two divergent strains (dark grey bars). BAPS group 1 is the largest group and contained all the remaining strains (light grey bars). Notably, neither the American strains nor the European strains formed particular clusters; this result is consistent with genetic exchange/migration between European and North American populations of *B. burgdorferi* s.s.. Remarkably, we did not detect any signals of admixture between the different BAPS groups. Taken together, these results indicate population structuring and trans-Atlantic exchange between Europe and the USA.

### Agreement between chromosomal phylogeny and MLST

**Figure 3:**
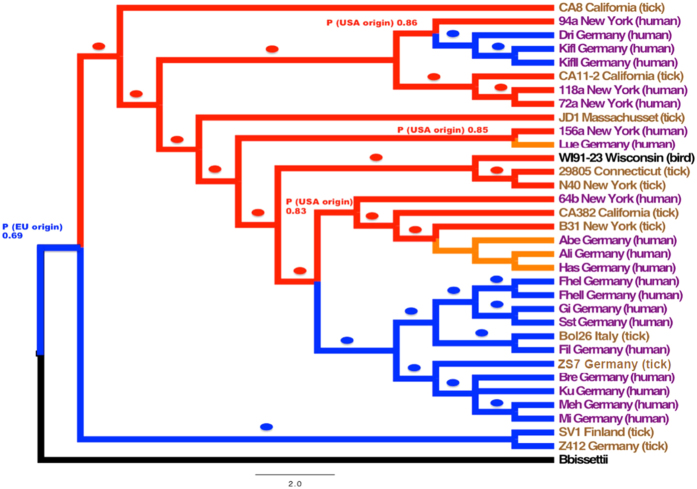
Bayesian Phylogeny of the 34 isolates. Bayesian phylogeny based on SNPs was not affected by recombination. The color-coding of the tips reflects the biological source of the isolates and it is as follows: purple, human isolates and brown tick isolates. The color of the branches describes the results of the ancestral state reconstruction analysis; blue reflects a European origin, whereas red implies USA origin. The little ellipses show those cases where the marginal posterior probability (MPP) of a European origin (blue) or North American origin (red) was higher than 0.95; cases where the MPP was lower than 0.95 are shown in the tree. The orange branches describe those strains that, although they were collected in Germany, the true geographic origin is uncertain. We used FigTree v1.4.0 to transform the branches (cladogram) for clarity; the tree without transformation is available as Supplementary Figure 1. The scale bar shows the estimated number of substitutions per SNP.

We used phylogenetic analysis to provide additional insights into the population structure of *B. burgdorferi* s.s.. Given the distorting effects of recombination on phylogenetic analysis, we first identified the recombination events between genomes via BRATNextGen^35^; this software implements a Bayesian analysis for detecting recombination events in whole-genome sequences. This analysis suggested that 8,321 out of 68,158 (~12%) of the SNPs reside in regions of the genome that have undergone recombination. Using the remaining SNPs not affected by recombination (the SNP alignment is available upon request), we constructed both Bayesian and Maximum Likelihood (ML) phylogenies, which showed very similar topologies; for simplicity only the Bayesian phylogeny is presented (Fig. 3, Supplementary Figures 1 and 2). All three groups identified by BAPS show very strong node support (posterior probabilities ranged from 1.00 to 0.79).

Given the tree topologies of our Bayesian and ML phylogenies, we wanted to ensure that this result was not due to long-branch attraction (LBA); the wrong grouping of two or more long non-sister branches in a tree due to homoplasies^36^. Specifically, we wanted to corroborate that the position of the divergent group was not a result of LBA and, hence, we conducted two exploratory analyses. The rationale of the long-branch extraction strategy (our first approach) is to take out the long branches - the ones allegedly causing the LBA - one at time, and see whether this disturbs the groupings present in the tree^37^. Therefore, we constructed one ML phylogeny without *B. bissettii* and another without SV1 and Z41293 (see methods). Notably, the groupings in the trees were hardly affected when either *B. bissettii* or SV1 and Z41293 were removed (Supplementary Figure 4) and the trees were very similar to those in our initial Bayesian and ML phylogenies; thus LBA does not seem to affect our phylogenetic reconstructions. However, to provide additional independent support, we also carried out a partition analysis where the idea is to eliminate the fast-evolving sites, the ones most contributing to LBA. Thus, we created a phylogeny based on the SNP alignment without the fast-evolving sites (see methods); this phylogeny shows identical groups to the Bayesian and ML phylogenies and was very similar to the phylogenies from the previous analysis (see Supplementary Figure 5). These two exploratory analyses convincingly show that LBA is not affecting our data and that the position of the divergent group with respect to the outgroup is not an artifact.

Our tree suggested that different genotypes are likely to have been introduced several times into the same area. For instance, PLue, is genetically distant from PDri, PKifI and PKifII, even though all four strains were collected in Munich. The same applies for the USA strains; for example, although strains CA8 and CA382 were both collected in California, these strains are located on distant branches of the tree. Furthermore, this phylogeny strongly suggests that intercontinental exchanges might have occurred from the USA to EU. By way of illustration, PAbe, PAli, and PHas (all from Germany) cluster within a group that also includes B31 and CA382 (from New York and California, respectively). Another example is 156a, which was collected from New York and clusters with PLue, a German isolate. Given the topology of our tree, at least four trans-Atlantic migrations might have happened, all of them within BAPS group 1. Our phylogenetic analysis is in good agreement with the pattern of genetic relatedness among the strains that had been previously shown using MLST based on eight chromosomally located housekeeping genes^20^ (Supplementary Figure 3) suggesting that MLST provides a good approximation of strain relatedness. Furthermore, the phylogenetic analysis gives additional support to the suggestion that *Borrelia* populations are genetically structured, have experienced trans-Atlantic exchange and that SV1 and Z412 are the most divergent strains within the *B. burgdorferi* s.s. species. As most of our strains originated from human patients, we used the MLST data to compare the strains analysed here with tick-derived strains in the MLST database (Supplementary Figure 6). All but two STs (i.e. ST1 and ST3, which have so far been only found in human patients) are also found in the European tick vector. The majority of STs found in ticks and humans in the USA differ from those found in Europe, the exception being ST1 and ST3.

### Geographic origin

To infer the geographic origin of *B. burgdorferi* s.s., we conducted an ancestral state reconstruction analysis using SIMMAP^38^; the results of this analysis are summarized in Fig. 3. This figure shows that a European origin is moderately more probable than a North American origin for the ancestor of all the *B. burgdorferi* s.s. strains (marginal posterior probability (MPP) of EU origin 0.69) but is highly likely for the group composed of the two divergent strains included in BAPS group 2 (MPP of EU origin 0.95). The directionality of subsequent movements is, however, not straightforward. According to our analysis, we were able to detect several trans-Atlantic exchanges and most of them seem to go from the USA to Europe. For instance, if only the three strains PAli, PAbe and PHas are considered in the analysis – a tight cluster of strains from Munich – a European origin is very plausible (MPP of EU origin 0.999). However, if the closest related strain (namely B31 from New York) to those three strains is included, the situation is reversed and now a North American origin is much more likely (MPP of USA origin 0.978, Table 3); this implies an introduction from the USA into Europe. Similarly, given that the ancestor of the group composed of 156s and PLue seems to have been in the USA (MPP of USA origin 0.85, see Fig. 3), PLue may reflect a recent introduction to Europe from the USA. The MLST Sequence Types (ST) were ST1 for B31, PAli, PAbe, PHas and ST3 for PLue. In this context it is noteworthy that both STs have frequently been found in *I. scapularis* ticks in the USA (but not in *I. ricinus* ticks in Europe) further supporting an American origin of the respective clades. Interestingly, STs that are found in European ticks (e.g. ST20, ST24) form their own cluster within the phylogeny and these STs have not been described in either patients or ticks in the USA (Supplementary Figure 6). Thus, the ancestral state reconstruction analysis suggests a moderately more likely European origin for the original population and also clarifies the probable directionality of some of the trans-Atlantic exchanges.

## Discussion

In this study we sequenced 17 European *B. burgdorferi* s.s. strains isolated from human patients and from a tick via NGS. We also included previously sequenced strains into our analyses (to produce a data set of 34 strains) to obtain a better understanding of the relationship of strains between Europe and the USA and the origin of *B. burgdorferi* s.s.. Importantly this dataset, although limited in extent, includes both human and tick isolates from both Europe and the USA (see Tables 1 and 2); without considering the outgroup, 11 isolates (~33%) came from ticks and 13 isolates (~39%) were collected in the USA. Even though the newly sequenced isolates included in the study originated mainly from German patients, they do represent the most frequently found MLST STs of *B. burgdorferi* s.s. in Europe, which have been found in ticks in various regions such as Latvia, France and UK (39,40, www.pubmlst.org/borrelia/).

We focused on the main linear chromosome because it is more likely to reflect the core genome^28^ and may be less prone to horizontal transfer than plasmid-encoded genes^27^,^33^,^41^. However, we found that a significant amount of the SNPs (12%) were located on regions inferred to be affected by recombination. This result was not unexpected as previous studies suggested recombination to mutation rates of 3:1 in *B. burgdorferi* s.s., although this ratio was found in plasmids rather than the main linear chromosome^33^. However, more recent population genomics studies using the main linear chromosome and two plasmids of European strains of *B. burgdorferi* s.s. suggested recombination to mutation ratios of 1.7^ ^42. These data reinforce the view that recombination is one of the forces creating novel combinations of alleles within this species.

Strain clustering in phylogenetic analysis correlated only poorly with geography. Notably, of three well-differentiated genetic groups, one comprised both European and American strains, supporting the idea of trans-Atlantic exchange^33^. In addition, phylogenetic analysis showed that strains from Europe are nested within the USA clades suggesting that at least four intercontinental migration events have occurred in that direction (i.e. from the USA to Europe). The routes and vehicles of these exchanges are unknown and deserve further investigation (see below) though our data suggest that some European patients may have acquired their infection in North America. One ancestral migration might have occurred at a more basal position of the tree resulting in the split between BAPS1 and BAPS2 clades, an event that gave rise to the USA population. Apart from this, our phylogenetic analysis demonstrates that multiple introductions, with different genotypes, might have occurred into the same geographic area. This is also supported by the BAPS analysis, as we found that strains from sites that are in geographic close proximity belong to different BAPS groups. In accordance with this, no admixture was detected between the BAPS groups, not even between BAPS groups 2 and 1, both of which have *B. burgdorferi* s.s. strains. Therefore, besides geography with intercontinental exchange, additional unknown factors probably operate to generate population structuring.

Whether the divergence of the two strains from BAPS group 2 reflects also differences in ecology is currently unknown. Strains related to Z41293 (here abbreviated Z412) have been found in *I. ricinus* ticks in Switzerland and France^20^, but further ecological information on these strains is not available. Z41293 and related strains have been used to determine the species threshold for *B. burgdorferi* s.l.^39^,^43^. Strain Z41293 and related strains were considered “borderline” *B. burgdorferi* s.s.^43^ because DNA-DNA hybridization studies found that this strain showed 74 percent similarity and a Tm of 2 °C to strain B31. Using MLST, strain SV1 also falls within the borders of *B. burgdorferi* s.s. species. As the former strains served to set the threshold for species delineation, in our view it is problematic to designate them as a new species^44^. Despite the contentious status of Z41293 and related strains, these divergent strains have so far only been detected in Europe, but not in North America supporting the idea of a European origin of *B. burgdorferi* s.s..

We conducted an ancestral state reconstruction analysis to infer the geographic origin of the species and it suggests that the ancestor of all the strains is moderately more likely to have had a European origin than a North American origin. This relates to the fact that the most divergent group (BAPS group 2) has a probable European origin. In this context, we would like to point out that, in order to correct for the sampling bias (more European isolates), we employed an equal prior so that *a priori* both a European and a USA origin had an equal probability of occurrence. This finding combined with the fact that these strains have been found only in Europe further supports the notion that the seminal population originated in Europe as suggested previously by^20^. Our analysis also implies that after the origin of the primordial population, several trans-Atlantic exchanges have evidently occurred.

However, considering the position of *B. burgdorferi* s.s. in a whole species tree^7^, a number of potential alternative scenarios can be considered: A) the common ancestor of *B. burgdorferi* s.s. originated in Europe and following the split from the divergent clade (BAPS group 2), the resulting *B. burgdorferi* s.s. migrated to North America. Since then several trans-Atlantic migrations have occurred forming the European subpopulations and a North American subpopulation. B) Another possibility is that all *B. burgdorferi* s.s. evolved in North America. The divergent group migrated to Europe and died out in North America. C) *B. burgdorferi* s.s. evolved in North America and the divergent group still exists in North America but at very low frequency and has simply not been found in natural transmission cycles. It needs to be emphasized that the species tree shown by 7 was based on eight chromosomal housekeeping loci and may change if more genetic information is incorporated. We acknowledge that denser sampling from both Europe and the USA – especially of isolates from this divergent group – and time dating of population migrations is needed to be able to resolve these issues unambiguously. We also suggest that in order to resolve the phylogeography of *B. burgdorferi* s.s., other genospecies of the LB group of spirochetes need to be included into future phylogenetic investigations.

It is difficult to reconcile the population structure of *B. burgdorferi* s.s. with natural transmission cycles. Two different tick vector species maintain the transmission cycles in Europe and North America, namely *I. ricinus* and *I. scapularis*, respectively. Trans-Atlantic migration and mixing of tick vector species has not been reported suggesting that the Atlantic Ocean represents a barrier to migration/mixing of vector species^14^. Although *B. burgdorferi* s.s. can utilize many hosts as reservoirs including rodents, insectivores and birds^16^, none of these hosts is known to regularly migrate East-to-West or *vice versa* across the Atlantic. Whether sea birds may be involved in transcontinental migration of *B. burgdorferi* s.s.^45^ needs to be further investigated. We would like to highlight that strains of *B. burgdorferi* s.s. found in Europe - and showing the same ST as North American strains (ST1 and ST3) - have only been isolated from human patients (see below) (23,46, and this study), so humans are potentially the vehicle to transport strains of *B. burgdorferi* s.s. between continents. If this is the case, these strains are highly unlikely to enter natural transmission cycles as humans are not considered to contribute to the natural transmission of *Borrelia*^14^. Intriguingly, ST1 strains of *B. burgdorferi* s.s. are commonly found in ticks and patients in the USA, but they have not been reported from ticks in Europe (see pubmlst.org/borrelia/ database^46^). There are several possible scenarios that might explain the finding of such closely related strains to B31 in patients in Europe. First, these patients may have acquired their *Borrelia* infections while travelling in the USA. Second, such strains may be present at a very low prevalence in ticks in Southern Germany as ticks investigated for *B. burgdorferi* s.s. had been collected in Latvia, Scotland, France or Italy and this ST has not been found^47^,^48^. As pointed out before, *B. burgdorferi* s.s. is not very prevalent in Europe; LB species such as *B. garinii* or *B. afzelii* are much more common than *B. burgdorferi* s.s.^49^. In this scenario, the fact that B31-related strains are not found in ticks but only in patients might suggest a high potential for establishing an infection in humans. However, a discrepancy between tick and human isolates has been observed in the USA, not all STs isolated from human patients have been detected in ticks^50^ suggesting that some strains although rare in natural transmission cycles can be found in human patients.

The NGS data provided very robust phylogenies and it was interesting that the distribution of STs matched well with clades generated by MLST analysis. This suggests that MLST can be used as a good approximation to explore/investigate the relationship of *B. burgdorferi* strains, although some authors reported otherwise^28^. Interestingly and, in contrast to other bacterial pathogens, there was a limited amount of variation between strains belonging to the same ST. For example, strains that belonged to ST1 (PAli, PAbe, PHas) differed in only 63, 64 and 261 SNPs, respectively, to B31 on the main linear chromosome. In comparison, an ST (i.e. ST239) of *Staphylococcus aureus* strains comprising identical MLST types differed in more than 4,000 SNPs^51^. On the other hand, due to the high resolution provided by NGS, no two strains show the same genotype – not even FheI and FheII that were collected from the same patient at the same time. That was still true when only the non-recombinant SNPs were considered.

In conclusion, our study shows that population structuring and trans-Atlantic exchanges have occurred during the evolutionary history of *B. burgdorferi* s.s. and may still occur today. However, we do acknowledge that more data are required to infer migration routes and transport vehicles. Importantly, it seems that some European patients may have acquired their infection in North America. Thus, our study clearly gives a demonstration of the utility of NGS platforms for studying the evolutionary history of vector-borne bacterial pathogens, especially for those where genetic variation is low.

## Materials and Methods

### The data set and genome sequencing

We analyzed 17 strains that were previously typed to be *B. burgdorferi* s.s. and were available at the German National Reference Centre for *Borrelia* (Table 1). *B. burgdorferi* s.s. is less common in Europe than in North America (13% of all European species in the MLST database). The strains included in the study were chosen to account for the genetic variation found by MLST in European *B. burgdorferi* s.s. to attempt to limit phylogenetic discovery bias^52^. The MLST STs included were ST20, ST21, ST24, ST284 and ST27, which represent the most common STs in *Ixodes ricinus* ticks from Germany, Latvia, UK, France and human patients (see PubMLST.org/datbase/borrelia/). ST27 represents a divergent strain, which was used to determine the species threshold in *Borrelia*^39^. ST1 was frequently found in human patients and was included to determine its relationship to strains found in North America^23^. Strains were isolated between 1985 and 2010 from patients and one *I. ricinus* tick. Details are given in Table 1. *Borrelia* cultures grown under standard conditions^53^ were used for DNA extraction via a Maxwell® 16 genomic DNA purification kit (Promega, Germany). Following DNA quantification, libraries were prepared according to the Nextera DNA sample preparation guide (Qiagen, Germany). The samples were diluted to a concentration of 50 ng per microliter and “tagmented” by simultaneously fragmenting DNA using transposomes as provided by the manufacturer and adding adapters. After tagmentation, samples having adapters on both ends underwent 5 PCR cycles to amplify the product and to add index primers. The resulting libraries were then validated using an Agilent 2100 Bioanalyzer (Agilent, Germany). Paired end sequence reads (250 bp) were generated using an Illumina Miseq platform (Illumina, San Diego CA, USA). We used the B31 and SV1 main linear chromosomes (B31, GenBank accession number NC 001318; SV1, GenBank accession number NZ ABJZ00000000.2) as reference. We concentrated on the main chromosome because it most likely represents the core genome for *Borrelia*^27^ which is best suited to infer the evolutionary history of strains. For *de novo* assembly, reads were quality checked and filtered using FastQ Trimmer (window size 10, step size 2, Q score 20)^54^, kmernator (k-mer size 31) and velvetoptimizer^55^ implemented in the Galaxy platform^56^,^57^ provided by the Gene Centre, LMU, Munich, Germany. The following parameters were used in velvetoptimiser: start Hash length 35, end hash length 85, no. of threads 4, k-mer optimization metric N50. Resulting contigs were mapped onto the reference chromosomes with Burrows-Wheeler Aligner (BWA)^58^ and re-arranged and sorted using the Mauve Contig Mover algorithm^59^ which is within the Mauve Genome Alignment Software^60^. The reference chromosomes were B31 for 16 strains and SV1 for strain Z41293. Contigs that did not align to the main linear chromosome were discarded. Some of the publicly available genomes are in a draft status and we therefore used the Mauve Contig Mover to order the contigs; in such cases B31 was used as the reference genome. We used progressive Mauve^32^ to align the chromosome genomes of our newly sequenced strains as well as those from the previously published strains. We employed the default parameters and a SNP file was created; 92 colinear sub-alignments (LCBs) were found, but only eight of them contained DNA conserved in all the strains. An SNP alignment was created which is available upon request. For comparison with the MLST scheme used by^20^ and^50^, sequence data of the eight housekeeping loci (*clpA, clpX, nifS, pepX, pyrG, recG, rplB, uvrA*) were extracted from whole genome sequences if not already available from the MLST database hosted at the University of Oxford (PubMLST.org).

### Neighbor net and detection of recombination

We employed SplitTree4 software on the SNP alignment to construct a Neighbor Net^61^, the uncorrected P distance was used. We use the BRATNextGen software^35^ to identify the recombination events exclusively in the eight LCBs that contained DNA conserved in all the strains. We used similar settings as those used by Castillo-Ramirez *et al.*^4^. A p-value of less than 0.01 was set to determine the significance of the recombinant segments, this was done via a bootstrap test with 100 replicates.

### Maximum Likelihood and Bayesian phylogenies

We constructed phylogenies by means of PhyML (Maximum Likelihood; ML)^62^ and MrBayes^63^. The multiple alignment on which the phylogenies were run, contained only SNPs that were located in the eight conserved LCBs and that were not present in regions affected by recombination. We used the GTR model and, because we only considered SNPs, we did not include a proportion of invariable sites. Base frequencies as well as the relative substitution rates between them were calculated by maximizing the likelihood of the phylogeny. We set the GTR model, allowing gamma-distributed rate variation across sites. We ran the analysis for 8,000,000 generations, sampling every 1000 generations −25% were discarded as burn-in. To ensure that two runs had converged we checked that the average standard deviation of the split frequencies were below 0.01.

### Bayesian Analysis of Population Structure (BAPS)

The mixture and admixture analyses were implemented through BAPS 5^34^. To conduct the population mixture analysis we selected the “Clustering of individuals” module. To properly evaluate the number of genetically diverged groups, we ran the analysis several times with different values for the maximum number of genetically diverged groups; these values ranged from 2–20. We chose the “Admixture based on mixture clustering” module to implement the population admixture analysis. The minimum population size was set to 2; we used 100 iterations to calculate the admixture coefficient for the individuals and the number of reference individuals from each population was set to 200. Finally, 20 iterations were used to calculate the admixture coefficient for the reference individuals.

### Ancestral Reconstruction Analysis

The ancestral state reconstruction analysis was carried out by means of SIMMAP^38^. The ancestral state reconstruction module was employed on the trees that were derived from a Bayesian analysis conducted with MrBayes. The geographic source of the strains was coded as 1 (Europe) and 0 (USA) and we configured the models as follows: for the bias parameter we selected an equal prior (1/k) – this was in order to not bias our analysis given the overrepresentation of European strains - and for the rate parameter we specified a gamma distribution with an alpha of 1.25, beta of 0.25 and k equal to 90.

### Detecting long-branch attraction (LBA)

We used two strategies to detect LBA. First, we conducted a long-branch extraction approach^37^ in which the branches that are potential candidates for causing LBA are extracted one at time. If LBA is the problem this should affect the clustering pattern in the remaining taxa. Therefore, we constructed two new ML phylogenies: one without the outgroup and another without strains SV1 and Z41293 that are located on the second longest branch (see Supplementary Figure 4). As a second strategy, we conducted a partition analysis, in which the fast-evolving sites are discarded as these mostly contribute to LAB. For this, MEGA6^64^ was used to estimate position-by-position rates under the GTR model employing a discrete Gamma distribution (with 5 categories) to model the rate differences across the SNPs. Then, a new alignment was created that included only the SNPs with slower relative evolutionary rates than the two fast evolving categories from the discrete Gamma distribution. A ML phylogeny was made on this alignment (see Supplementary Figure 5).

## Additional Information

**How to cite this article**: Castillo-Ramírez, S. *et al.* Trans-Atlantic exchanges have shaped the population structure of the Lyme disease agent *Borrelia burgdorferi* sensu stricto. *Sci. Rep.* **6**, 22794; doi: 10.1038/srep22794 (2016).

## Supplementary Material

Supplementary Information

Dataset 1

## Figures and Tables

**Table 1:** Newly sequenced strains.

Strain Name	Abbreviation	Diagnosis	Year of isolation	Origin	MLST ST
PMeh	Meh	Arthritis	1996	Garmisch	ST20
PBre	Bre	EM*	1988	Munich	ST20
PDri	Dri	EM	1988	Munich	ST24
PFil	Fil	EM	1985	Munich	ST284
PSst	Sst	EM	1993	Tübingen	ST21
PAli	Ali	EM	1994	Regensburg	ST1
PLue	Lue	EM/multiple	1999	Munich	ST3
PFheI	FheI	Lymphom	2010	Munich	ST21
PFheII	FheII	Lymphom	2010	Munich	ST21
PGl	Gl	NB^**§**^	1993	Bayreuth	ST21
PHas	Has	NB	1992	Ulm	ST1
PKu	Ku	NB	1996	Bad Mergentheim	ST20
PMi	Mi	NB	1994	Munich	ST20
PAbe	Abe	NB	1997	Munich	ST1
PKifI	KifI	Skin/chest	1988	Munich	ST24
PKifII	KifII	Skin/knee	1988	Munich	ST24
Z41293	Z412	N/A - Tick isolate		Germany	ST27

*B. burgdorferi* s.s. strains newly sequenced, symptoms caused, year of isolation, and geographic origin within Germany. Reads have been submitted to the NCBI Sequence Read Archive (SRA) under accession number SRP051650

*Erythema migrans.

^**§**^Neuroborreliosis.

N/A = not applicable.

**Table 2:** Publicly available isolates.

Isolate (GenBank accession)	Geographic origin	Biological source	MLST ST
**B31** (AE000783.1)	New York, USA	*I. scapularis* (tick)	ST1
**CA382** (CP005925.1)	California, USA	*I. pacificus* (tick)	Nd
**64b** (ABKA02000001.1 - ABKA02000006.1)	New York, USA	Human	ST59
**ZS7** (CP001205.1)	Germany, EU	*I.ricinus* (tick)	ST20
**Bol26** (ABCW02000001.1 - ABCW02000005.1)	Italy, EU	*I.ricinus* (tick)	ST332
**WI91-23** (ABJW02000001.1 - ABJW02000031.1)	Wisconsin, USA	Bird	ST228
**29805** (ABJX02000001.1 - ABJX02000038.1)	Connecticut, USA	*I. scapularis* (tick)	ST12
**N40** (CP002228.1)	New York, USA	*I. scapularis* (tick)	ST19
**JD1** (CP002312.1)	Massachusetts	*I. scapularis* (tick)	ST11
**156a** (ABCV02000001.1)	New York, USA	Human	ST4
**CA-11.2A** (ABJY02000001.1 - ABJY02000014.1)	California, USA	*I. pacificus* (tick)	ST333
**118a** (ABGI02000001.1 - ABGI02000008.1)	New York, USA	Human	ST34
**72a** (ABGJ02000001.1 - ABGJ02000006.1)	New York, USA	Human	ST14
**94a** (ABGK02000001.1 - ABGK02000009.1)	New York, USA	Human	ST18
**CA8** (ADMY01000001.1 - ADMY01000007.1)	California, USA	*I. pacificus* (tick)	Nd
**SV1** (ABJZ02000001.1 - ABJZ02000005.1)	Finland, EU	*I.ricinus* (tick)	ST414
***B. bissettii***DN127* (ABJZ02000001.1 - ABJZ02000005.1)	California, USA	*I. pacificus* (tick)	NA

We used the BorreliaBase database (http://borreliabase.org) to access these chromosomal genomes. This database gives direct access to the GenBank webpages that contain the files for the chromosomal DNA for each isolate. The GenBank accession numbers are in parenthesis.

*This is a closely related species that was used as an outgroup.

**Table 3:** Ancestral State Reconstruction Analysis.

Group	Prob(USA origin)	Prob(EU origin)
All *B. burgdorferi* s.s. strains	0.311892	0.688108
BAPS group 2 (SV1 and Z412)	0.049205	0.950795
PLue and 156a	0.849954	0.150046
PAli, PAbe, PHas and B31	0.977758	0.022242
PAli, PAbe, PHas	0.000002	0.999998

Posterior probabilities of a European or an American origin for all the different groups.
