# Supplementary material for: Trans-Atlantic exchanges have shaped the population structure of the Lyme disease agent Borrelia burgdorferi sensu stricto
Source: Sci Rep. 2016 Mar 9;6:22794. doi: 10.1038/srep22794 (PMC4783777; doi:10.1038/srep22794)

Trans-Atlantic exchanges have shaped the population structure of the Lyme disease agent *Borrelia burgdorferi* sensu stricto

Castillo-Ramírez S., Fingerle V., Jungnick S., Straubinger R. K., Krebs S., Blum H., Meinel D.M., Hofmann H., Guertler P., Sing A., Margos G.

Supplementary Table 1 – Genome sequencing features

Median Coverage, read numbers and association with MLST of *B. burgdorferi* s.s. strains

Strain Name	Abbreviations+	Diagnosis	median coverage	Total number Reads/ % B31 chromosome coverage	MLST ST
PMeh	Meh	Arthritis	70	658,228/98	20
PBre	Bre	EM*	211	824,291/99	20
PSst	Sst	EM	42	538,366/98	21
PDri	Dri	EM	349	713,770/99	24
PFil	Fil	EM	250	585,070/99	284
PAli	Ali	EM	419	3,083,916/99	1
PLue	Lue	EM/multiple	34	717,740/90	3
PFheI	FheI	Lymphom	341	845,626/99	21
PFheII	FheII	Lymphom	347	899,117/99	21
PGl	Gi	NB§	148	1,116,854/98	21
PHas	Has	NB	43	332,678/97	1
PMi	Mi	NB	48	844,536/85	20
PKu	Ku	NB	357	843,761/99	20
PAbe	Abe	NB	205	632,500/99	1
PKifI	KifI	Skin/chest	284	668,738/99	24

PKifII	KifII	Skin/knee	399	860,144/99	24
Z41293	Z412	*Ixodes ricinus*	47	662,322/95	27

+Abbreviations used for the phylogenetic and BAPS analysis

*Erythema migrans

§Neuroborreliosis

Supplementary Table 2 – Characteristics of the LCB used for the phylogenetic analysis 1 In parenthesis are the nucleotide diversity values for each LCB.

LCB	Length	Number of SNPs	% of B31 chromosomal genome aligned
6	4569	309 (0.0112)	0.48 %
7	216991	16144 (0.009)	23.01 %
9	257617	16837 (0.0089)	25.55 %
10	40738	3085 (0.0083)	4.23 %
11	163044	11769 (0.00856)	17.31 %
12	187750	13940 (0.0087)	19.77 %
13	22166	1587 (0.0081)	2.35 %
14	57999	4406 (0.0093)	6.15 %

Supplementary figure 1 - Bayesian Phylogeny of the 34 isolates

Bayesian phylogeny without branch length transformation. The color coding of the tips reflects the biological source of the isolates and it is as follows: purple, human isolates and brown tick isolates. The color of the branches describes the results of the ancestral state reconstruction analysis; blue reflects a European origin, whereas red implies USA origin. The orange branches describe those strains that, although they were collected in Germany, the true geographic origin is uncertain. The scale bar shows the estimated number of substitutions per SNP.

Supplementary figure 2 – Maximum Likelihood phylogeny of the 34 isolates

The phylogeny was constructed with PhyML (see methods). Color coding as in Supplementary figure 1. The scale bar shows the estimated number of substitutions per SNP.

Supplementary figure 3 - Bayesian Phylogeny showing the ST assignation

Bayesian phylogeny with the ST for most of the strains. The color-coding of the tips is as in Supplementary figure 1. The numbers by the nodes show the posterior probabilities for the different clades. The scale bar shows the estimated number of substitutions per SNP.

Supplementary figure 4 – Long-branch extraction approach

ML phylogenies for the long-branch extraction approach. A) ML phylogeny without the two strains from the divergent group; B) ML phylogeny without the outgroup. The color-coding of the tips is just to show that similar groupings are obtained with both phylogenies. The scale bar shows the estimated number of substitutions per SNP.

Supplementary figure 5 – Partition analysis

ML phylogeny based on SNP alignment without the fast evolving sites. Here the color-coding of the tips is the same as Supplementary figure 4 and clearly demonstrates that similar groups are yielded with this analysis. The scale bar shows the estimated number of substitutions per SNP.

Supplementary Figure 6 – *B. burgdorferi* sensu stricto multilocus sequence analysis (MLSA) sequence types (ST)

Borrelia burgdorferi sensu stricto multilocus sequence analysis (MLSA) sequence types (ST) found in human patients and tick vectors in Europe and the USA. Note that there is limited overlap in STs between the two continents; only ST1 and ST3 are present in both regions but have been found only in humans in Europe. The data were downloaded from the MLSA database at www.pubmlst.org/borrelia/. ST20, ST21, ST23 and ST24 match *B. burgdorferi* s.s. strains recently described by Jacquot et al. (2014), i.e. IPT69, IPT2, ZS7; IPT19; IPT137, respectively). Contigs for IPT141, IPT49 and IPT77 were downloaded from GenBank and typed to be ST24 (or closest match ST24 as for IPT141 and IPT77 only short fragments of the MLST sequences for some genes were recovered). These authors isolated a number of *B. burgdorferi* s.s. strains from a forest location in France but no strain was found to be identical to B31 (ST1) or ST3.

Supplementary figure 1

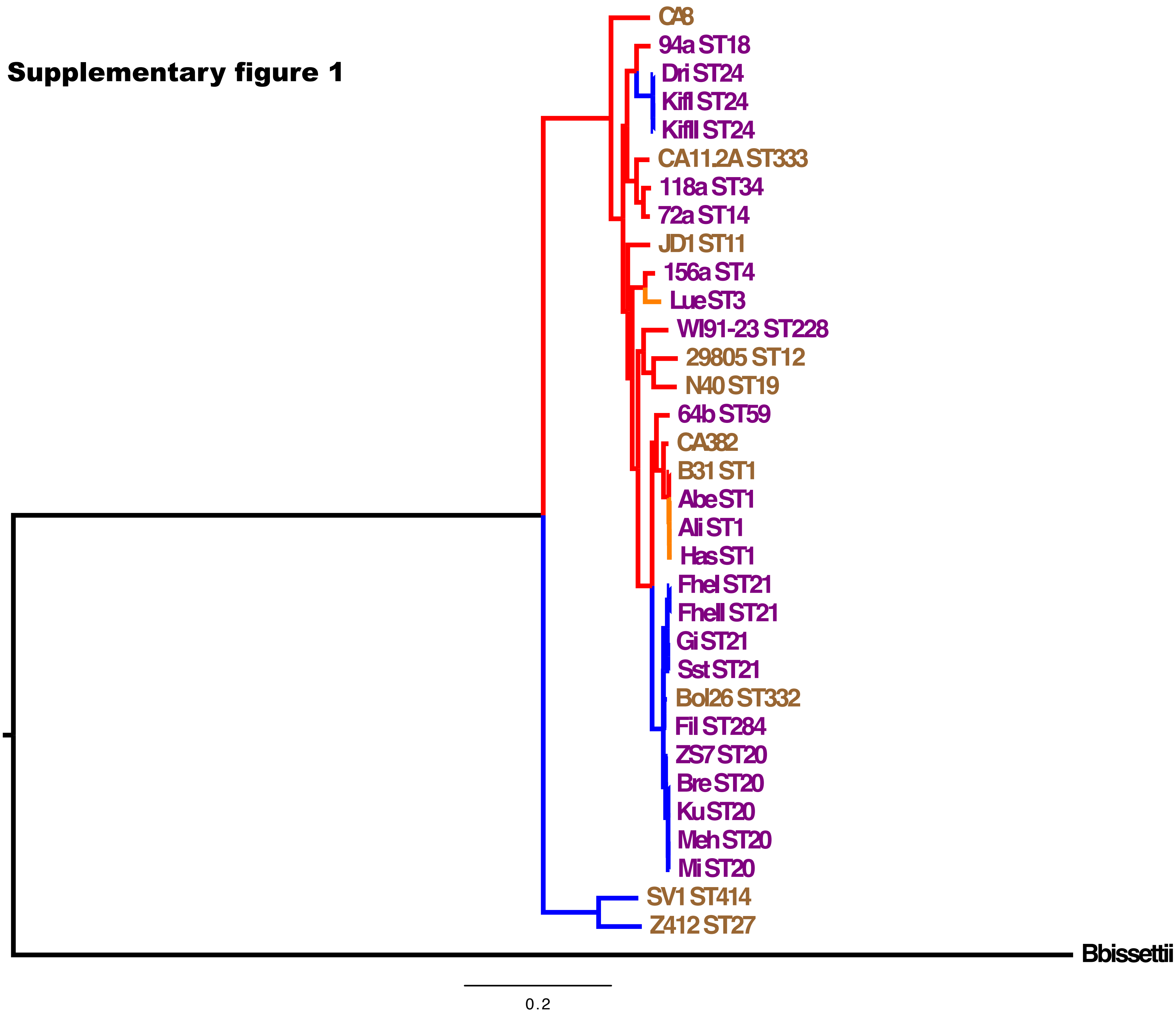

Supplementary figure 2

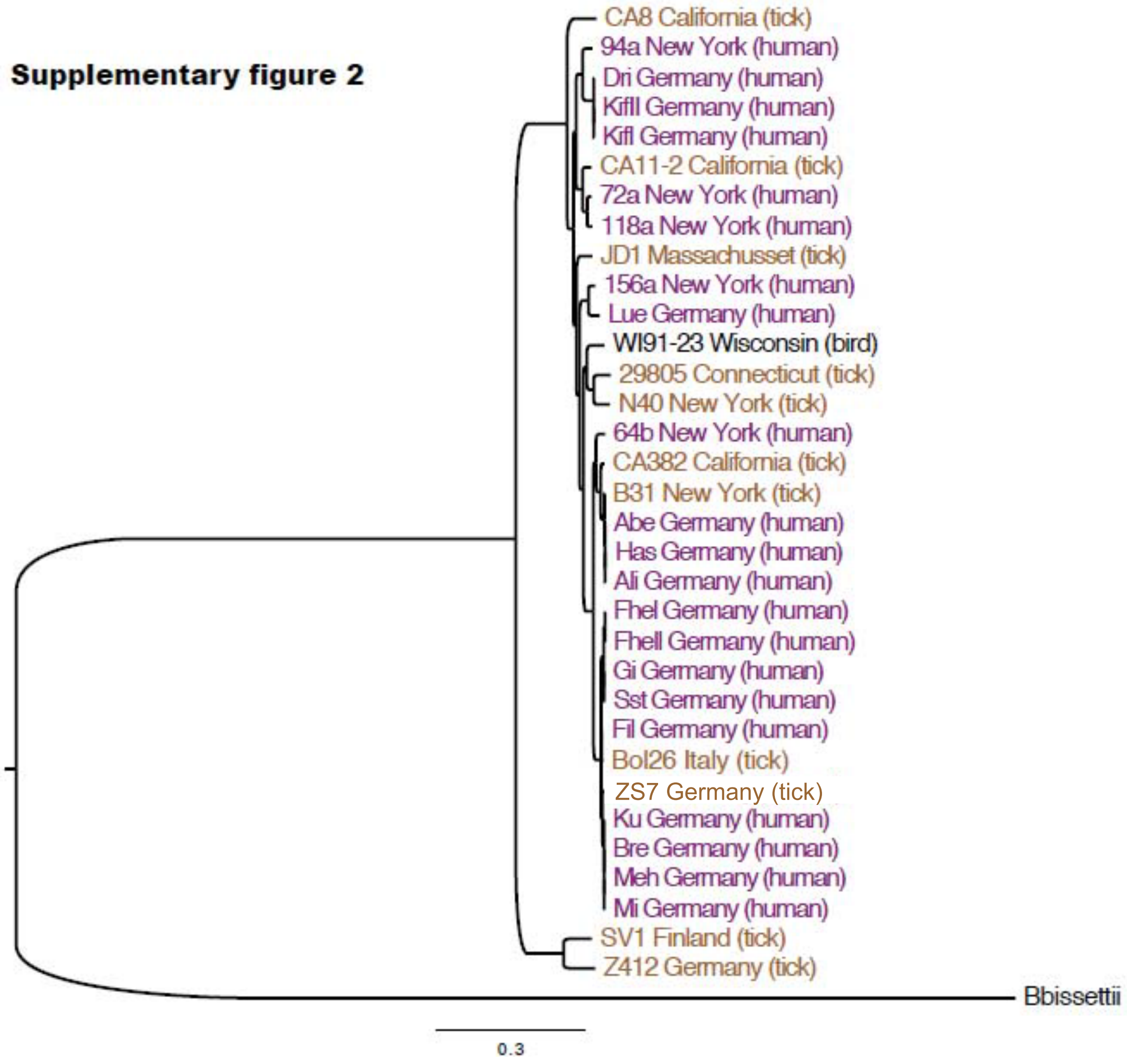

Supplementary figure 3

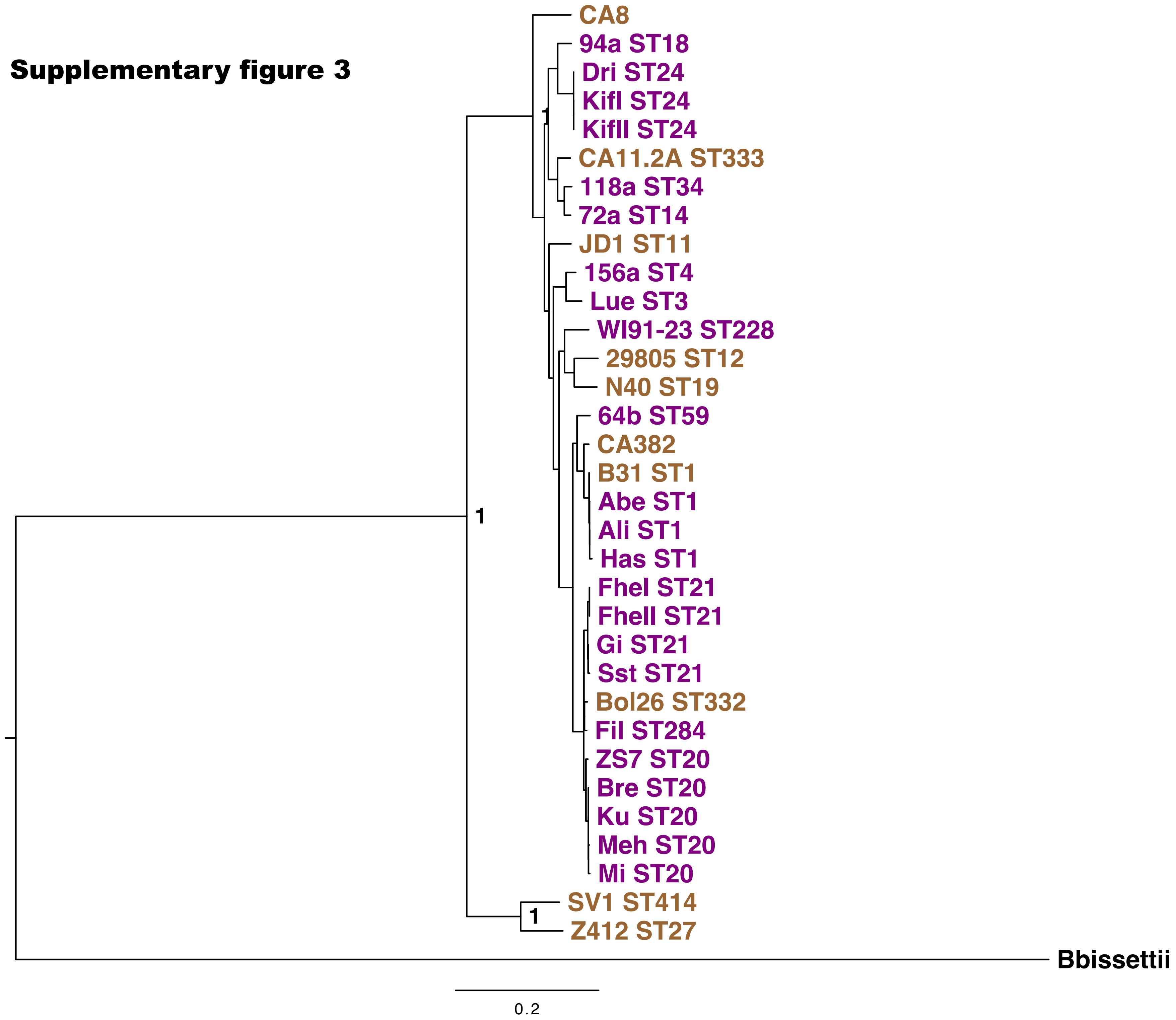

Supplementary figure 4

A) No SV1 and Z412

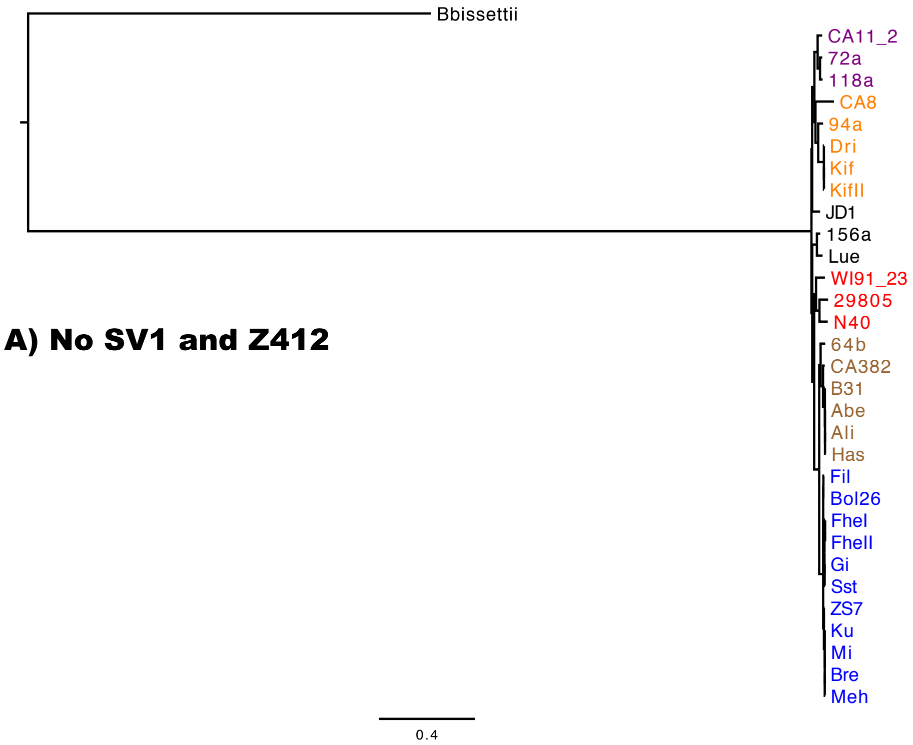

B) No Bbissetti

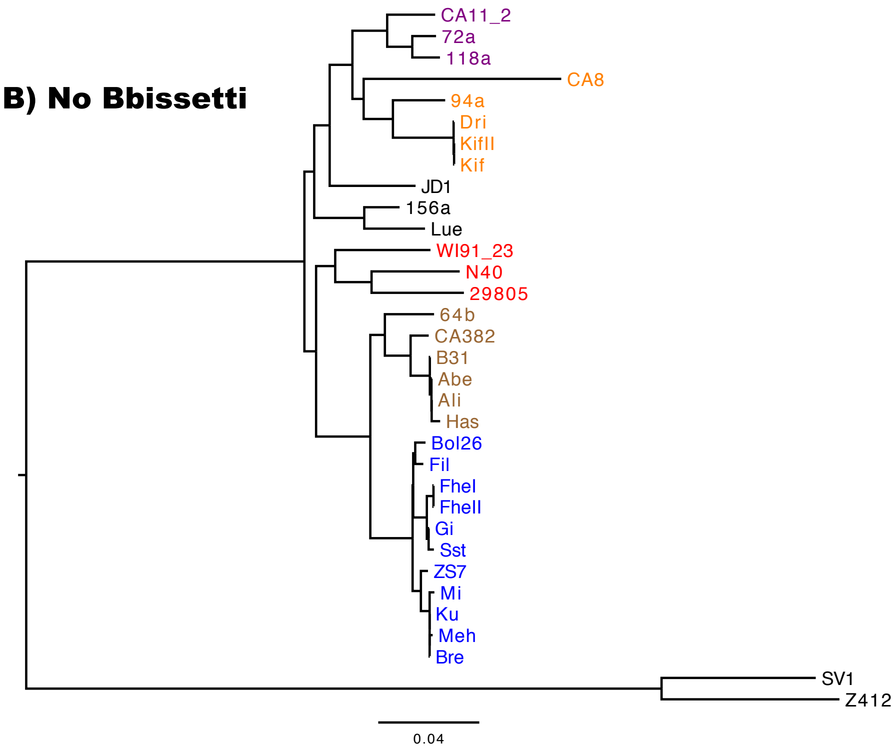

Supplementary figure 5

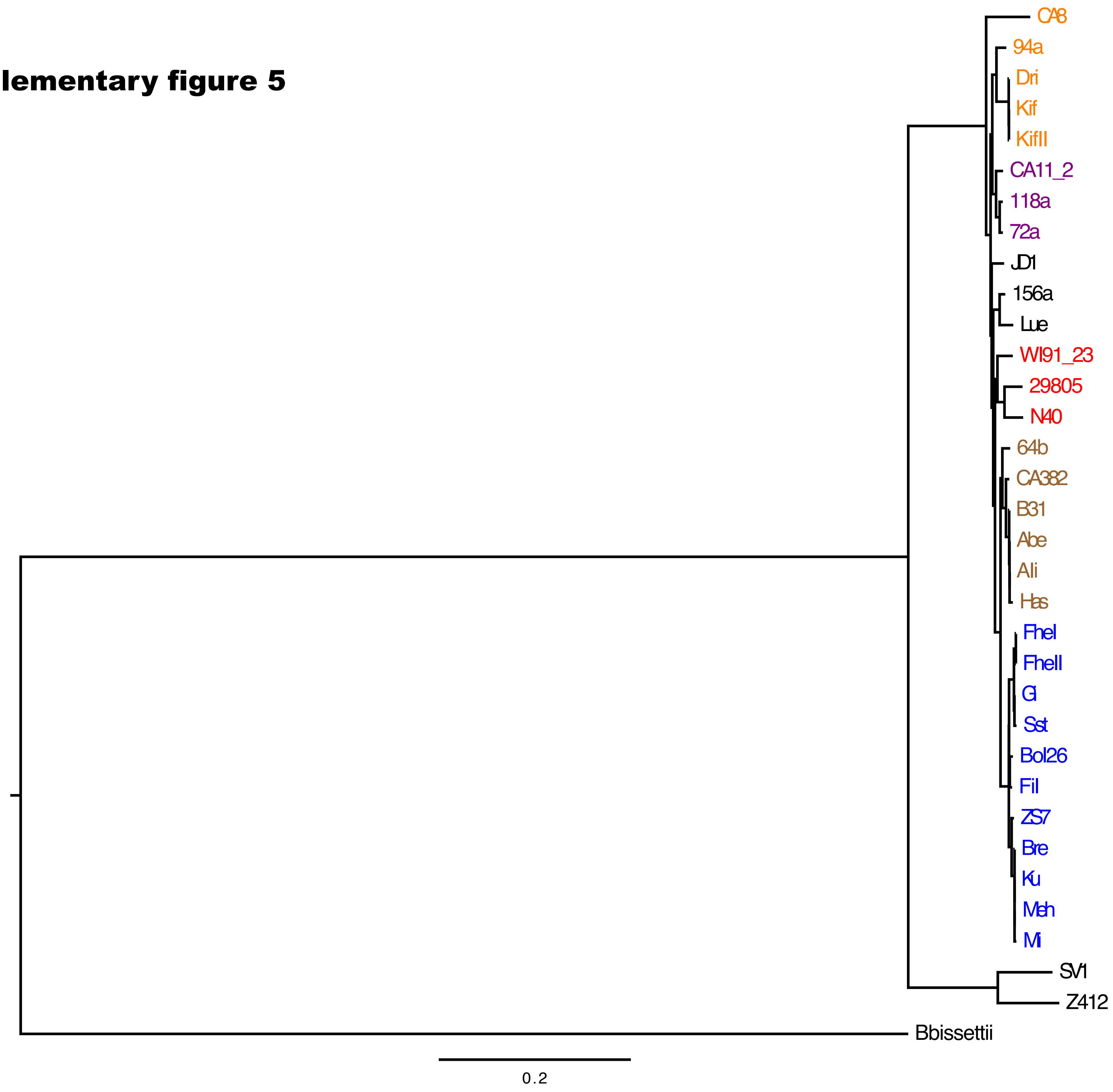

Supplementary figure 6

Borrelia burgdorferi sensu stricto sequence types in Europe and USA

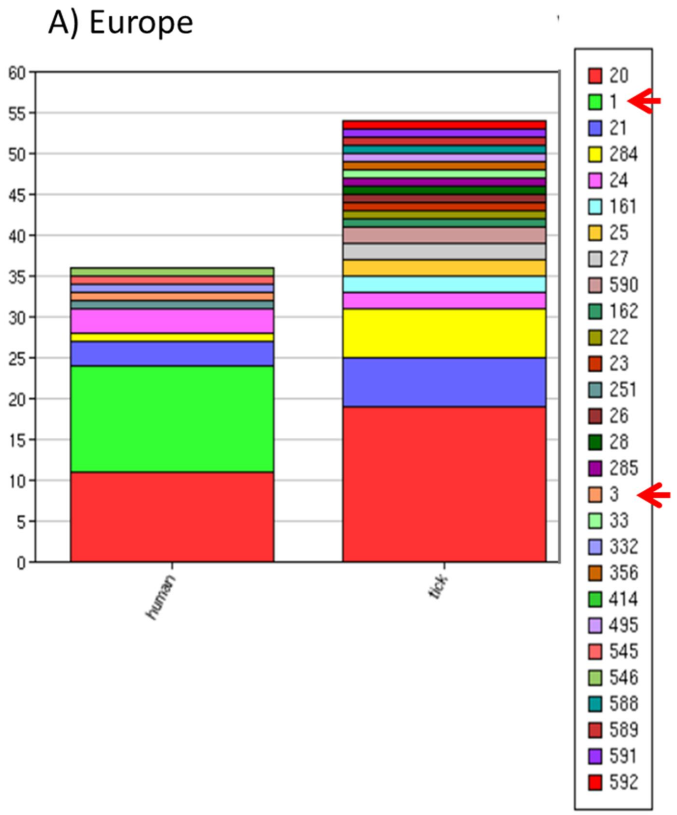

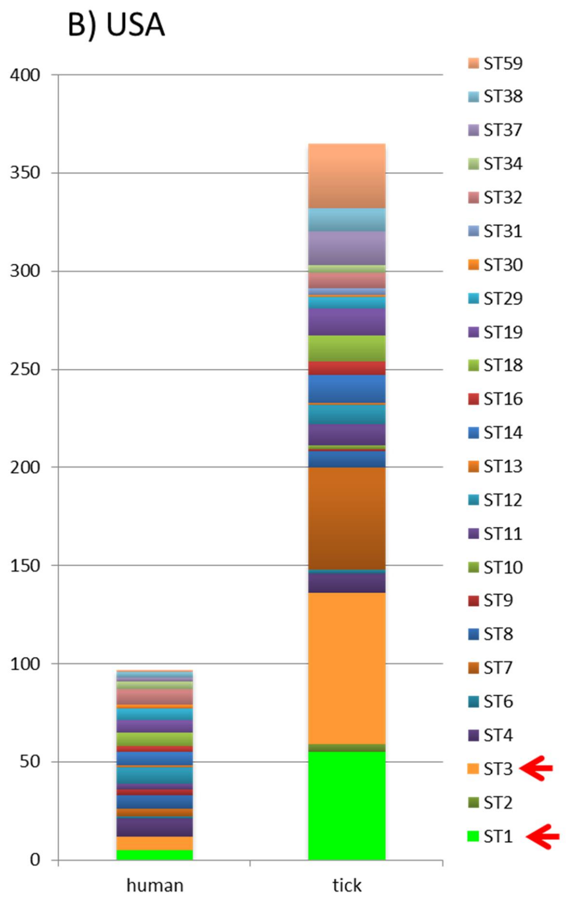

Supplement: Supplementary Information [file srep22794-s1.pdf]
